# Fractures of the Hamate Bone: A Review of Clinical Presentation, Diagnosis and Management in the United Kingdom

**DOI:** 10.7759/cureus.73839

**Published:** 2024-11-17

**Authors:** Muhammad Arham Sahu, Abdullah Tahir, Muhammad Ashal Sahu, Adam Varachia, Haseeb Khawar, Usman Ahmed

**Affiliations:** 1 Trauma and Orthopaedics, Hereford County Hospital, Wye Valley NHS Trust, Hereford, GBR; 2 School of Medical Sciences, University of Manchester, Manchester, GBR; 3 Trauma and Orthopaedics, University Hospitals Nottingham NHS Trust, Nottingham, GBR; 4 Trauma and Orthopaedics, Worcestershire Royal Hospital, Worcestershire Acute Hospitals NHS Trust, Worcester, GBR

**Keywords:** carpal fractures, hamate, hamate fracture, hand trauma management, hook of hamate

## Abstract

Injury to the hamate can result in significant functional impairment and a negative impact on quality of life. These injuries, in general, occur very infrequently, and in the setting of an increasingly ageing population and more patients presenting post falls, clinicians are at risk of either misdiagnosing or failing to diagnose patients who sustain hamate fractures.

This review finds that hamate fractures can occur through acute trauma or chronic repetitive stress. These injuries are often missed resulting in a delay to management. Common presenting features of hamate fractures include ulnar-sided palmar tenderness and paraesthesia in the ulnar nerve distribution, loss of grip strength and pain on swinging a gripped object. In examination, the hamate “pull test” is a highly sensitive test for hamate fractures. CT scan of the carpal bones is shown to be the gold standard investigation for the diagnosis of such injuries. Initial management of hamate fractures in the United Kingdom is in line with the National Institute of Health and Care Excellence (NICE) guidelines that involves immobilisation with a suitable splint and sling for comfort, adequate analgesia, rest and elevation. Specific management of hamate fractures is divided into conservative and surgical options; however, this review identifies the need for further research into which form of management is superior.

## Introduction and background

The hamate is the ulnar-most bone located within the distal row of carpal bones, neighboured by the capitate bone radially. It has a palmar bony process that projects from its body towards the hypothenar eminence, known as the hook of hamate [1]. This prominence is most often greater than 7mm in height and carries anatomical significance as it forms a point for attachment of multiple structures: the hook tip attaches the pisohamate ligament, the flexor retinaculum, and forms the ulnar part of the carpal tunnel. The body of the hook of hamate forms the radial border of Guyon’s canal and also acts as a pulley for the fourth and fifth digit flexor tendons [2-4].

Approximately 20% of all fractures treated in the emergency department are related to the hand, of which carpal bone fractures make up approximately 8% [5,6]. The most common types of carpal bone fractures involve the scaphoid and triquetrum bones, which together comprise almost 90% of all carpal bone fractures [7]. In comparison, fractures of the hamate bone are relatively rare, occurring in 2-4% of all carpal bone fractures [4,8]. The hamate bone can sustain a fracture at either its body or its hook; fracture of the hook of hamate is the most common type of fracture for this carpal bone, and injury to this bony process can result in significant functional impairment and negative impact on quality of life for patients [8,9].

With an increasingly ageing population and more patients admitted to hospitals post-falls, fractures of the carpus can be easily missed as there are usually more significant reasons surrounding the fall. Given the paucity in occurrence of this kind of injury, clinicians can misdiagnose or fail to diagnose patients who present with it. On average, the time to diagnosis from the point of initial injury is nearly six months, which, in turn, results in delay in appropriate management and consequent loss of function of related soft tissue structures [10-12]. It is therefore imperative that hospital doctors are proficient at diagnosing and treating these injuries.

This review aims to collate and summarise information from the existing evidence base on how patients with fractures of the hamate can present, as well as the approach to diagnosis and the options for management.

## Review

Clinical presentation

The most common symptoms that patients with a hamate fracture can present with include tenderness over the ulnar side of the palm, loss of grip strength and pain when swinging a gripped object [12]. This type of injury is typically more common in athletes and can occur both acutely from blunt force trauma, such as falling onto an outstretched hand or catching a ball travelling at high speed, or chronically over weeks from repeated shear stress placed on the hook through prolonged gripping of objects such as a bat, club or racquet [1,2,13,14]. Athletes who play golf, tennis or cricket are particularly at increased risk of sustaining this injury during the swinging motion of the gripped object, as forces placed on the hook of hamate are near maximal load [15]. The affected hand can vary depending on the type of sport the patient participates in, with tennis players sustaining the fracture most commonly in their dominant hand whilst other athletes such as golfers and cricketers injuring their non-dominant hand in contrast [16].

**Figure 1:**
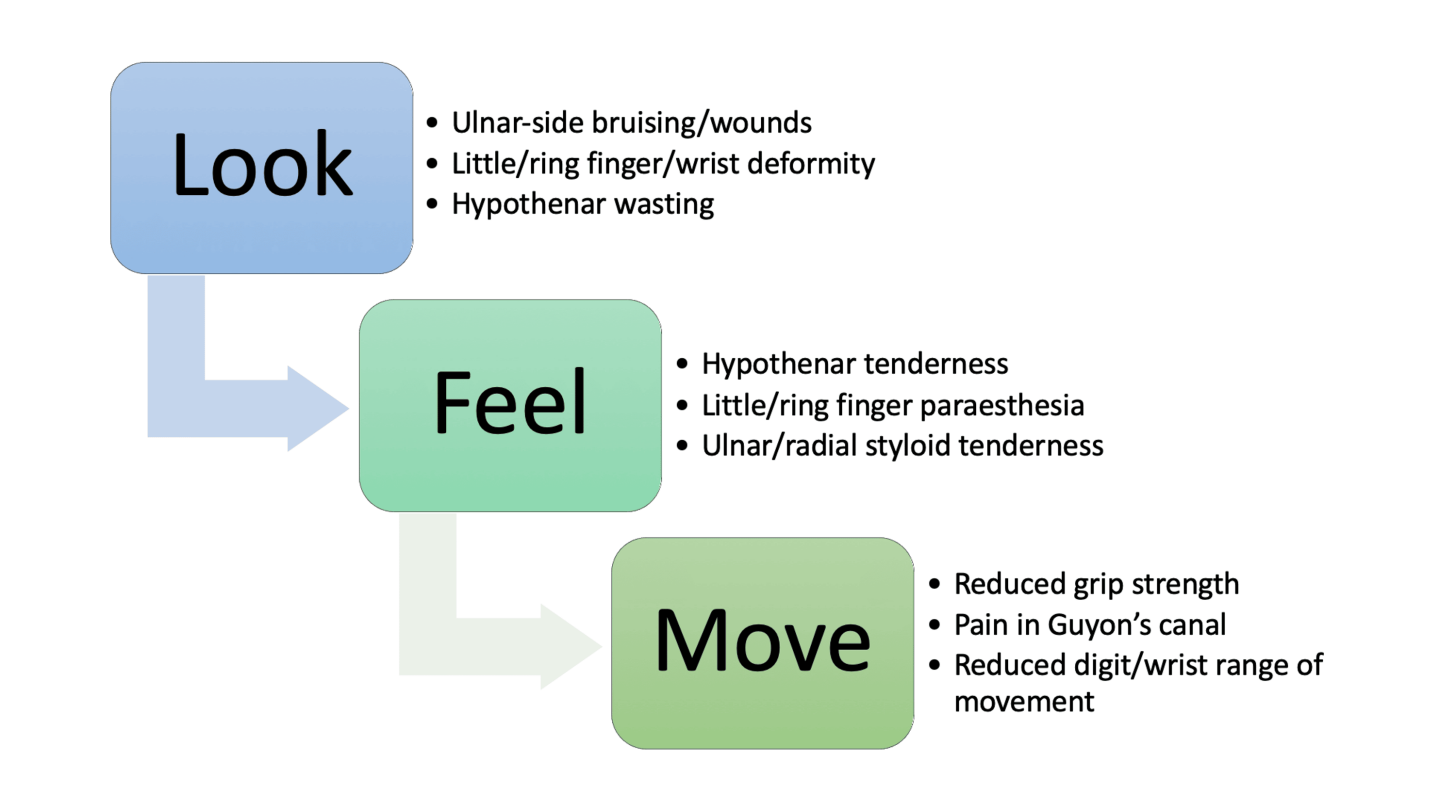
Hand examination findings suggestive of underlying injury to the hamate. This figure has been created using Microsoft Office 365 by author Muhammad Arham Sahu.

**Figure 2:**
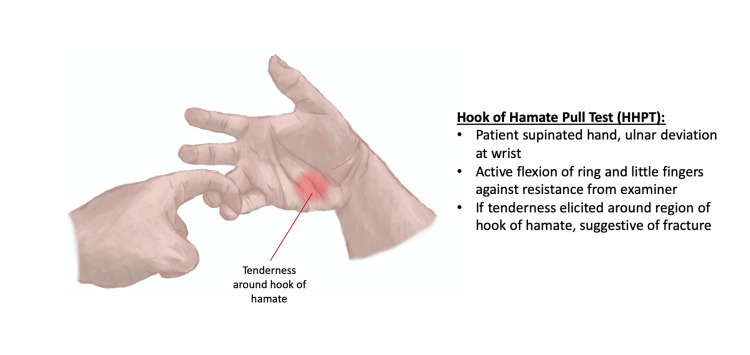
Explanation of how to perform HHPT. This figure has been illustrated using Procreate and annotated using Microsoft Office 365 by author Muhammad Arham Sahu. HHPT, hook of hamate pull test

Clinical examination can be divided broadly into three categories: inspection, palpation and movement, colloquially known as “look, feel and move” (Figure 1). In a patient with a suspected hamate fracture, a number of signs can be found with varying frequencies of occurrence. On inspection, evidence of bruising or wounds over the ulnar side of the hand, in addition to finger or wrist deformity, may be indicative of underlying hamate injury. Atrophy of the hypothenar eminence may be noted in patients with older injuries of the hamate that may have previously been missed [17]. Tenderness on palpation, particularly in the area of the hypothenar eminence around the hook of hamate, can be suggestive of injury to this structure. Ring and little finger paraesthesia elicited through palpation is a sign of ulnar nerve palsy which can be associated with hamate fractures [13]. Trauma causing a fracture of the hamate can often also cause simultaneous fractures to other bones of the affected upper limb, especially the scaphoid bone and distal radial bone, and therefore tenderness on palpation of either radial or ulnar styloid processes can be considered a positive examination finding [18,19]. Repeated tapping of the hypothenar eminence which elicits altered sensation of the ulnar nerve as it passes through Guyon's canal is suggestive of hamate fracture [10,20]. On movement, the clinician will assess range of movement and power across flexion and extension at the wrist and digits. Weakness in grip strength, either due to neurological origin or secondary to pain, is another finding suggestive of fracture to the hamate. A diagnostic examination technique, the hook of hamate “pull” test (Figure 2), has also been shown to have a high sensitivity in detecting fractures of the hook [21].

Hamate fracture classification

**Figure 3:**
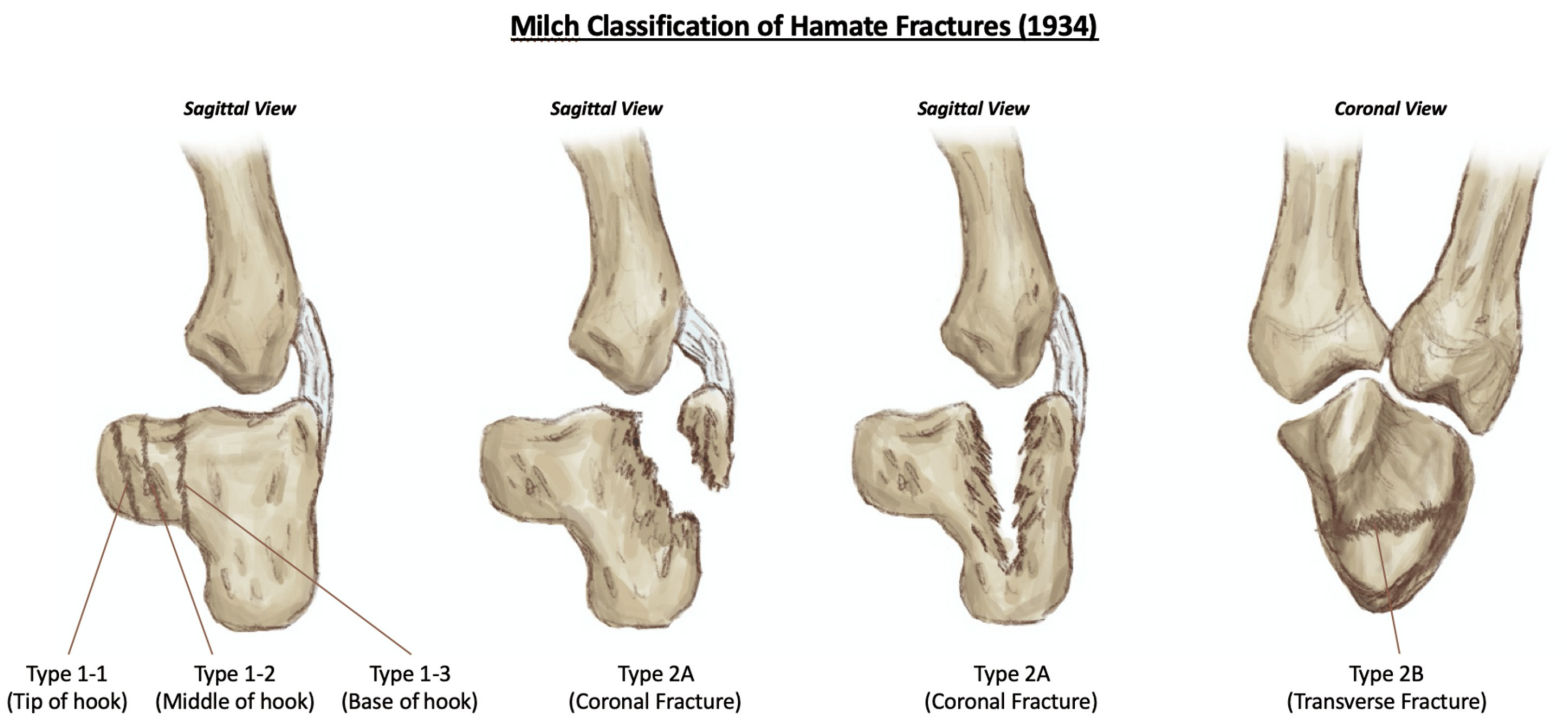
Illustration of the Milch classification of hamate fractures (1934). This figure has been illustrated using Procreate and annotated using Microsoft Office 365 by author Muhammad Arham Sahu.

Classification of hamate bone fractures (Figure 3) was first proposed by Milch in 1934, which divided fractures into two types: type 1, which involves the hook of hamate and is the more common type, and type 2, which involves the body of the hamate and subdivided into type 2A and type 2B, based on if the body of the hamate is fractured in the coronal plane or the transverse plane, respectively [22]. Hook of hamate fractures, Milch type 1, were then further divided into three main subtypes depending on the location of the fracture line on the bony process: type 1-1, which is a fracture of the tip of the hook, typically caused by avulsion, type 1-2, which is a fracture of the middle portion of the hook, and type 1-3, which is a fracture of the base of the hook and is the most common of all three subtypes [14,15,23].

Diagnostic investigations

A number of imaging modalities can be considered to aid in the diagnosis of suspected hamate fractures, namely plain radiographs, computed tomography (CT) scans and magnetic resonance imaging (MRI). Plain radiographs of the wrist in a posterior-anterior (PA) view generally have been reported to be an unreliable way of diagnosing these fractures [24,25]. Occasionally, on PA views of the carpal bones, a “ring” sign can be demonstrated which is the hook of the hamate being superimposed over its own body; if this ring is disturbed, it can be suggestive of a hook of hamate fracture [26]. Carpal tunnel view radiographs are useful in detecting type 1-1 and type 1-2 fractures; however, obtaining this view can prove technically challenging as it requires patients to dorsiflex their wrist, which may not be tolerated in the setting of a carpal bone fracture [27].

Plain radiographs do not always demonstrate a clearly visible fracture, particularly minimally displaced type 1-3 fractures. In patients in whom clinical assessment is suggestive of a hamate fracture, obtaining a CT scan despite negative plain radiographs can confirm the presence of a fracture [28]. CT scan of the carpal bones has been shown to be the most useful imaging modality for detecting hamate fractures, with a higher diagnostic accuracy in comparison with plain film radiographs [1,29].

MRI can demonstrate bone oedema signals in response to trauma, which can identify hamate fractures in patients who otherwise may present with normal radiographs [12]. MRI is also useful in providing information on surrounding vasculature and soft tissues that may have their signals altered as a result of avascular necrosis of the hook of hamate through traumatic injury, thereby revealing the presence of a fracture [12]. Through this, MRI has been able to identify chronic stress-related fractures of the hook of hamate in a number of reported cases [30].

Management

General management for all patients who present with any acute traumatic injury to the wrist includes provision of an adequate splint such as a futura splint or below elbow backslab, alongside a sling for elevation, as per the NICE guidelines [31]. Patients should be encouraged to rest, apply a cold compress and keep the injured limb elevated to allow for resolution of oedema. Gentle active movement of the fingers should be also encouraged to prevent stiffness.

Hamate fractures can be managed either conservatively or surgically. Conservative management is typically reserved for non-displaced fractures and involves cast immobilisation for a number of weeks, with benefits of avoiding risks associated with surgical intervention; however, evidence demonstrates generally poor outcomes for patients treated in this way, and acutely discovered fractures when treated conservatively with cast immobilisation between six weeks to four months had a non-union rate of approximately 24-83% [18,32-34]. Not all non-unions become symptomatic, but those that did, proceeded to undergo surgical management, which yielded resolution of symptoms with better overall outcomes [32]. Generally, conservative management of hook of hamate fractures have a higher non-union rate when compared with surgical management [10]. For hamate body fractures, those which are minimally displaced without adjacent joint malalignment have been successfully managed conservatively [35,36]. A drawback of conservative management is the varied compliance of patients who strictly adhere to recommendations for remaining non-load bearing; those who are less compliant tend to experience suboptimal outcomes as placing load confers risk of long-term pain and loss of function [37].

Surgical management of hook of hamate fractures can be achieved either through excision of the hook itself or through fixation, although there is controversy surrounding which arm of surgical management is superior. Surgical treatment is typically preferred for patients with symptomatic, displaced acute hook of hamate fractures or for those patients who fail a trial of conservative management [38]. The outcomes for surgical intervention, irrespective of excision or fixation, will vary according to patient factors, needs and requirements; on average, patients managed surgically returned to normal function and activity within 13 weeks post-operatively, with younger patients generally returning quicker [12,32]. Both excision and fixation will achieve resolution of patient symptoms, with lower rates of non-union compared to conservative management; however, excision is performed more commonly than fixation as it has been shown to have lower rates of post-operative pain and a quicker return to normal function/activity for patients [16,33,39]. Excision of the hook of hamate does, however, have drawbacks as loss of the hook will weaken of the pulley function of the ring and little fingers, which results in weakened power grip, and there is a higher risk of associated ulnar nerve dysfunction [39,40]. Surgical fixation provides the benefit of maintaining pulley function and preserving more grip strength compared to excision; however, patients have a higher rate of post-operative pain with a longer return to normal function [8,39].

As with hook of hamate fractures, surgical treatment is recommended for hamate body fractures, where there is significant displacement or instability, particularly if carpometacarpal joints become unstable [41,42]. Instability of these fractures has been suggested to be a consequence of the pull from extensor carpi ulnaris [42]. Hamate body fractures typically are treated with either closed reduction and percutaneous fixation (CRPF) or with open reduction internal fixation (ORIF), with the majority being treated using ORIF or a combination of the two techniques [43-45]. Surgical treatment using ORIF has been shown to have successful outcomes with regards to restoration of range of motion, grip strength and resolution of pain [35,45].

## Conclusions

This review of literature surrounding hamate fractures shows that these are rare injuries which can occur both acutely and through chronic repetitive stress. They are easily misdiagnosed or missed due to their infrequent occurrence and result in a delay to appropriate management and debilitating consequences. Typically, this type of injury occurs in athletes, namely those participating in golf, cricket or tennis. Given the delay to diagnosis, and some cases reported as asymptomatic non-union, there may likely be under-reporting of the actual frequency of occurrence of hamate fractures as some patients may not ever present.

CT scan of the carpal bones has been shown to be the golden standard for diagnosis of these fractures, as it can identify them in patients with otherwise normal plain radiographs. MRI has some value in identifying fractures associated with chronic stress to the hook.

Management of hamate fractures can be performed conservatively, or surgically through excision if the fracture involves hook of hamate, or fixation. Conservatively managed fractures were shown to have a higher rate of non-union compared with surgical management, with some patients developing symptomatic non-union and needing to ultimately proceed to surgical management. Published evidence does not clearly identify which option for surgical intervention, excision or fixation, is consistently superior for hook of hamate fractures. Benefits and drawbacks exist for each, but the reliability of these reported outcomes is questionable given that the majority of evidence comparing the two interventions is based on retrospective case analysis. These studies had relatively small case numbers and inclusion of patients with a range of ages, backgrounds, activities and mechanism of injuries, and both acute and chronic fractures. These factors are likely to influence the outcomes of each surgical option; therefore, it is difficult to make a fair comparison between each option and confidently state which one is superior. Similarly, for hamate body fractures, whilst the majority of the relatively low number of published evidence suggests that ORIF confers more favourable post-operative outcomes for patients, there is some considerable reporting of cases using operative techniques which blend together percutaneous wire fixation with ORIF and still maintain an excellent outcome for patients.

This review identifies the need for further research into which form of management, whether conservative or surgical, is superior. Furthermore, within surgical management, further studies are needed that explore which surgical management option is superior with regards to outcomes. Larger scale, prospective, randomised controlled trials would provide high quality evidence to inform clinical decisions regarding conservative or surgical management of hamate fractures, and, if surgical, then which type of surgery.

Lastly, for the clinician assessing patients with suspected a hamate fracture in the emergency setting, this review also draws attention to the importance of conducting a thorough examination, features of the history that should raise index of suspicion of a hamate fracture and the limitations of plain radiographs and subtle fractures that can appear normal on this form of imaging. Hamate fractures are generally rare injuries but often do not occur in isolation. Without timely intervention, they can lead to debilitating loss of function and a significant detrimental impact on patients. Prompt referral to the local fracture clinic or hand trauma unit, with early specialist orthopaedic review and subsequent follow-up, will reduce this negative impact and improve patient outcomes.
